# Surgical Correction of Sprengel’s Deformity in Children Using the Modified Green Technique: A Functional and 3D Motion Analysis Study

**DOI:** 10.3390/jcm14248941

**Published:** 2025-12-18

**Authors:** Philipp Scheider, Fabian Unglaube, Andreas Kranzl, Catharina Chiari, Sebastian Farr

**Affiliations:** 1 Department of Pediatric Orthopaedics and Foot and Ankle Surgery, Orthopaedic Hospital Speising, 1130 Vienna, Austria; catharina.chiari@oss.at; 2 Laboratory for Gait and Movement Analysis, Orthopaedic Hospital Speising, 1130 Vienna, Austria; fabian.unglaube@oss.at (F.U.); andreas.kranzl@oss.at (A.K.)

**Keywords:** Sprengel deformity, modified green procedure, upper extremity, shoulder girdle malformation, omovertebral bone, Cavendish classification, patient-reported outcomes, functional outcome, motion analysis

## Abstract

**Background/Objectives:** Consensus exists on the effectiveness of surgery in improving scapular position and appearance in Sprengel’s deformity, yet evidence regarding functional outcomes is limited. This study aimed to evaluate functional results of the modified Green procedure in children and to assess patient-reported outcome measures. **Methods:** Patients who underwent corrective surgery using the modified Green technique between 2006 and 2023 were analyzed. Demographic and treatment-specific parameters were collected. The clinical severity of the deformity was graded according to the Cavendish classification, and radiographic evaluation was performed using the Rigault classification. Therapeutic success with respect to mobility was determined by comparing pre- and postoperative abduction and elevation. Postoperative movement patterns of the upper limb were further evaluated using three-dimensional motion analysis. To quantify outcomes related to quality of life and functionality, standardized questionnaires were administered, including the Quick-DASH, the Shoulder Pain and Disability Index (SPADI), and the UCLA Shoulder Scale. **Results:** A total of 15 cases were included. The mean age at the time of surgery was 6.9 ± 4.0 years, with a mean follow-up of 4.8 ± 4.7 years (range, 0.6–17.7). Glenohumeral abduction improved to 90° in all cases, representing a mean gain of 8° (preoperatively: 82 ± 11°). Elevation improved by an average of 55° (preoperatively: 108 ± 28°; postoperatively: 163 ± 25°). At final follow-up, the mean Quick-DASH score was 10.7 ± 12.7, the mean SPADI score was 10.9 ± 12.1, and the mean UCLA Shoulder Scale was 31.9 ± 4.3, indicating excellent clinical outcomes. **Conclusions:** The modified Green procedure appears to be a safe and effective surgical technique for the correction of Sprengel’s deformity in children, demonstrating favorable outcomes in terms of mobility, function, and quality of life in this cohort.

## 1. Introduction

Sprengel’s deformity is the most common congenital malformation of the shoulder girdle and is characterized by a high-riding, hypoplastic, and malrotated scapula [1]. The deformity results from a disruption of the physiological caudal migration of the scapula during the 9th to 12th week of gestation, during which the inferomedial border of the scapula normally rotates medially and the glenoid tilts caudally [2,3,4]. In up to 60% of cases, a cartilaginous or osseous connection known as an omovertebral bone exists between the superior angle of the scapula and the spine [5,6,7]. Despite extensive discussion in the literature, the exact etiology and incidence of Sprengel’s deformity remain unknown [8,9]. The condition is frequently associated with other congenital anomalies, particularly Klippel–Feil syndrome and congenital scoliosis [10,11].

Depending on the severity of the deformity, clinical symptoms range from mild cosmetic concerns to significant limitations in scapulothoracic mobility. In advanced cases, glenohumeral abduction and elevation can be markedly restricted [1,12]. Various surgical techniques have been developed to treat Sprengel’s deformity, with the modified Woodward and Green procedures being the most commonly used and widely accepted [2,13,14,15]. Andrault et al. have highlighted improved visualization of the superomedial scapular angle and facilitated rotational correction as key advantages of the modified Green procedure [16]. However, no clear superiority of either technique has been demonstrated to date [17].

While the efficacy of these procedures in improving cosmetic appearance and scapular positioning is well established, there is a lack of data regarding functional outcomes and patient-reported measures assessing daily function, satisfaction, and quality of life following corrective surgery. The extent to which these invasive procedures—which involve detachment and repositioning of entire muscle groups—affect postoperative movement patterns remains unclear. To date, no studies have evaluated such effects using three-dimensional motion analysis.

The aim of this study was to assess the functional outcomes of the modified Green procedure for treating Sprengel’s deformity in children. Specifically, we sought to analyze postoperative movement patterns using 3D motion analysis and to identify correlations with patient- and treatment-specific parameters. Furthermore, we aimed to define patient-reported outcome measures with respect to upper limb function and patient satisfaction.

## 2. Materials and Methods

### 2.1. Study Design and Patient Selection

This retrospective, single-center study included patients who underwent surgical correction of Sprengel’s deformity using the modified Green procedure between 2006 and 2023 [13]. Ethical approval for this study was waived by the Ethics Committee of the City of Vienna due to its purely retrospective design. The study was conducted in accordance with the Declaration of Helsinki (2013 revision). Informed consent was obtained from caregivers for participation in the study.

Inclusion criteria were an age below 18 years at the time of surgery and a minimum clinical follow-up of more than 6 months. Patients aged over 18 or with incomplete documentation were excluded. Demographic data, treatment-related clinical parameters, and perioperative complications were recorded. Reporting of this study follows the STROBE guidelines (Supplementary Table S1).

### 2.2. Surgical Technique

The procedure begins with the patient in the supine position, followed by a prophylactic midshaft clavicular osteotomy to facilitate repositioning of the scapula and minimize traction-related neurovascular risk during the procedure. After the osteotomy, the periosteum is closed to promote bony healing. The patient is then repositioned into prone position, and a curvilinear incision is made over the medial third of the scapula.

**Figure 1:**
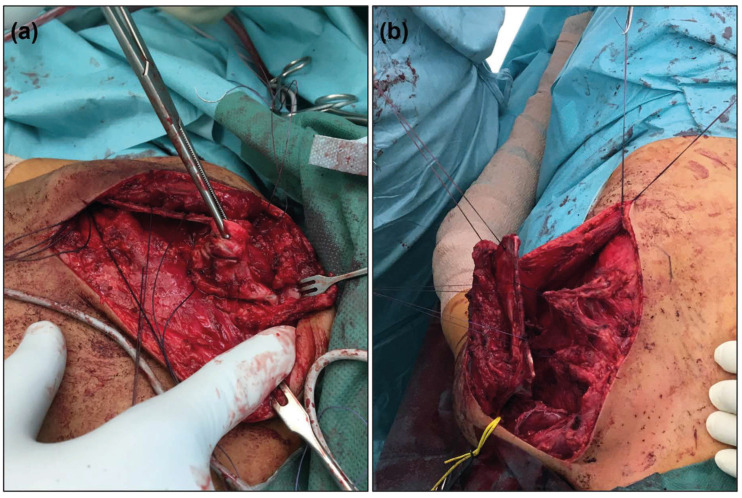
Selected key steps of the modified Green procedure. (**a**) Detachment of the fibrous or osseous connection to the spine. (**b**) Fully mobilized scapula prepared for repositioning.

The trapezius muscle is dissected to expose the scapula. The rhomboid muscles and the subscapularis are released en bloc, and a pocket is created beneath the latissimus dorsi. If a fibrous or osseous connection to the spine is present, it is resected (Figure 1). The supraspinatus and infraspinatus muscles are carefully detached from the scapula, allowing it to become fully mobilized, with attachment remaining solely at the glenohumeral joint. The transverse scapular ligament is then transected while preserving the suprascapular neurovascular bundle. The serratus anterior can be partially released. Prominent, hook-like bone edges, particularly in the cranial portion of the scapula, are resected. Drill holes are made in the scapula to allow reattachment of the released muscle groups. The scapula is then repositioned and caudalized into its anatomically corrected position and secured by looping a strong non-absorbable suture around the 10th rib.

Layered reattachment of the released muscles is performed in a newly established physiologic position: initially the supraspinatus, subscapularis, and serratus anterior; followed by the levator scapulae and rhomboid muscles; and finally the trapezius and latissimus dorsi. The procedure concludes with application of a Desault-type bandage for immobilization, maintained until clavicular healing (typically 4–6 weeks).

### 2.3. Clinical and Radiographic Assessment

Two classification systems were employed to evaluate the severity of the deformity. The Cavendish classification was used to assess the clinical appearance in the upright patient, ranging from grade 1 (no visible deformity) to grade 4 (shoulder asymmetry > 5 cm) [3]. For radiological assessment, the Rigault classification was applied, which categorizes scapular position based on the location of the superomedial scapular angle relative to the transverse processes on anterior–posterior radiographs [18]. Details of the classification systems are summarized in Table 1.

To assess shoulder mobility, glenohumeral abduction and elevation were measured during clinical examinations. Glenohumeral abduction refers to the range of motion of the shoulder in the frontal plane with the scapula manually stabilized by the examiner, enabling isolated assessment of glenohumeral joint mobility. In contrast, elevation describes shoulder movement in the frontal plane with unrestricted scapular motion.

Therapeutic success was assessed by comparing pre- and postoperative shoulder abduction and elevation, as well as changes in the Cavendish grade from baseline to final follow-up.

### 2.4. Functional Outcomes and Patient Satisfaction

Upper limb function was assessed using standardized patient-reported outcome measures, including the Quick-DASH (Disabilities of the Arm, Shoulder, and Hand), the Shoulder Pain and Disability Index (SPADI), and the UCLA Shoulder Scale [19,20,21].

The Quick-DASH consists of 11 five-point scaled questions, aggregated into a score ranging from 0 to 100 (0 = best function, 100 = worst).

The SPADI includes 13 items assessing pain and upper limb functionality in daily life, also resulting in a score from 0 (best) to 100 (worst).

The UCLA Shoulder Scale consists of five categories (pain, function, strength, range of motion, and satisfaction), with a maximum score of 35; scores above 27 indicate good to excellent outcomes.

Patient satisfaction regarding both functional and cosmetic outcomes was further assessed using numerical rating scales ranging from 1 (very dissatisfied) to 10 (very satisfied).

All patient-reported outcome measures were completed jointly by the children and their parents.

### 2.5. Three-Dimensional Motion Analysis

Postoperative movement patterns were evaluated using three-dimensional motion analysis of the upper limb. Following the protocol described by Van Andel et al., a total of 63 retroreflective markers (32 anatomical and 31 technical) were placed on the patient’s upper body [22]. Marker trajectories were recorded using a 3D motion capture system (17 cameras, 150 Hz; Vicon, Oxford, UK) to assess upper-extremity kinematics.

Data preprocessing was performed using Vicon Nexus version 2.16 and included trajectory reconstruction, labeling, gap filling, and filtering according to Woltring et al. [23]. Further analysis was conducted using a custom software tool (“Upper Limb Evaluation in Movement Analysis”) implemented in MATLAB 2022a (The MathWorks, MA, USA) [22].

Segment coordinate systems and joint centers were defined in accordance with the recommendations of the International Society of Biomechanics, as described by Wu et al. [24]. To assess upper limb kinematics following the modified Green procedure, the maximum active range of motion (ROM) of the shoulder and thorax was measured across all movement planes.

Five valid motion trials per side were conducted. Isolated active movements of the joints in the upper limb were performed (e.g., shoulder abduction) and the ROM was calculated as the range between the minimum and maximum amplitudes. Results were compared to a reference dataset previously collected in our laboratory for clinical and research purposes (*n* = 20; mean age: 29.3 ± 9 years; body height: 176 ± 9.8 cm; body weight: 73.4 ± 10.3 kg) [25]. The reference dataset consisted of healthy adults, as validated pediatric normative datasets for comprehensive three-dimensional upper-limb motion analysis are currently lacking. Fundamental shoulder movement patterns are considered comparable between children and adults.

If multiple postoperative motion analyses were performed for a patient, only the latest follow-up was used for evaluation. Only datasets with valid task execution were included, and no imputation of missing kinematic data was performed.

### 2.6. Statistical Analysis

Descriptive and inferential statistical analyses were performed using IBM SPSS Statistics, version 20 (IBM Deutschland GmbH, Ehningen, Germany). A *p*-value ≤ 0.05 was considered statistically significant.

Descriptive statistics for continuous variables were presented as mean (*M*), standard deviation (SD), median (Md), and interquartile range (IQR). For categorical variables, absolute frequencies (*n*) and percentages (%) were reported.

To assess postoperative improvements, paired comparisons of pre- and postoperative values were conducted. Normality of the differences was evaluated using the Shapiro–Wilk test. For abduction, the data were not normally distributed (*p* = 0.0009), so the Wilcoxon signed-rank test was applied. In contrast, elevation differences were normally distributed (*p* = 0.1943), allowing analysis via paired-samples *t*-test. Cavendish grades were also compared using the Wilcoxon signed-rank test, as the differences were not normally distributed (*p* = 0.0006).

Associations between interval-scaled, normally distributed variables were assessed using Pearson’s product-moment correlation coefficient.

For correlations between patient age at surgery and postoperative functional or subjective outcomes, Spearman’s rank correlation coefficient was used due to non-normal distributions (Shapiro–Wilk *p* < 0.05).

## 3. Results

### 3.1. Patient Characteristics

A total of 15 patients with complete documentation who underwent corrective surgery for Sprengel’s deformity using the modified Green procedure were included in the study. Of these, 10 patients (66.7%) were male (Table 2). The left side was affected in 8 cases (53.3%). The mean age at the time of surgery was 6.9 ± 4.0 years (range, 2.8 to 16.0 years). The mean postoperative follow-up was 4.8 ± 4.7 years (range, 0.6 to 17.7 years).

A structural connection between the scapula and the spine was observed in 9 cases (60.0%), consisting of an omovertebral bone in 6 patients (40.0%) and a fibrous connection in 3 (20.0%). All patients presented with a hypoplastic scapula. At least one associated congenital condition was present in 10 cases (66.7%). Scoliosis was observed in 8 patients (53.3%), and Klippel–Feil syndrome was confirmed in 4 (26.7%).

Radiographic grading according to Rigault revealed grade 3 deformity in 9 patients (60.0%) and grade 2 in 6 (40.0%). Based on the Cavendish classification, 11 patients (73.3%) were classified as grade 3, and 4 (26.7%) as grade 4.

### 3.2. Therapeutic Success and Range of Motion

Preoperative glenohumeral abduction averaged 82 ± 11 degrees (range, 60 to 90°) and showed statistically significant improvement to 90° at final follow-up in all patients (*p* = 0.007). Preoperative elevation averaged 108 ± 28 degrees (range, 85–170°) and improved by 55 ± 28 degrees postoperatively, reaching a mean of 163 ± 25 degrees (range, 90–180°). This improvement was statistically significant (*p* < 0.0001).

All patients demonstrated postoperative improvement in the clinical presentation of the deformity (*p* = 0.0001). At final follow-up, 14 patients (93.3%) were classified as Cavendish grade 1, and one patient (6.7%) as grade 2 (Table 3).

Hypertrophic scarring was observed in 6 cases (40.0%). One case of transient postoperative brachial plexus irritation was observed. Neurological symptoms resolved spontaneously within a short postoperative period without the need for additional intervention, and no persistent neurological deficits were present at final follow-up. No further complications occurred.

### 3.3. Functional Outcomes and Patient Satisfaction

At final follow-up, the mean Quick-DASH score was 10.7 ± 12.7 (range, 0 to 31.8), and the mean SPADI score was 10.9 ± 12.1 (range, 0 to 32.3), indicating minimal disability in most patients. The UCLA Shoulder Scale yielded a mean score of 31.9 ± 4.3 (range, 24 to 35), indicating generally good to excellent functional outcomes.

There was no statistically significant correlation between age and postoperative functional or subjective outcomes. Spearman’s rank correlation revealed a weak negative correlation between age and postoperative shoulder elevation (*r*_s_ = −0.298, *p* = 0.281), and between age and the UCLA Shoulder Scale (*r*_s_ = −0.327, *p* = 0.235). Conversely, weak-to-moderate positive correlations were observed between age and the Quick-DASH score (*r*_s_ = 0.318, *p* = 0.248) and the SPADI (*r*_s_ = 0.393, *p* = 0.147). These findings suggest a trend toward better outcomes in younger patients, although none of the correlations reached statistical significance.

Patient satisfaction was high across both functional and aesthetic domains. On a numeric rating scale from 1 (very dissatisfied) to 10 (very satisfied), mean satisfaction scores were 8.9 ± 1.6 (range, 6 to 10) for shoulder function and 8.9 ± 1.4 (range, 6 to 10) for cosmetic appearance.

### 3.4. Three-Dimensional Motion Analysis

All patients completed the standardized motion tasks of the 3D movement analysis protocol. Due to compliance issues in task execution, data from one patient had to be excluded.

Three-dimensional motion analysis revealed a reduction in active abduction/elevation on the affected side, with a mean value of 103.6 ± 38.1 degrees, compared to 134.0 ± 26.0 degrees on the contralateral side and 137.5 ± 10.6 degrees in the normative reference group. This corresponds to a postoperative reduction in abduction/elevation range of motion of approximately 30 degrees.

The lower values observed in 3D motion analysis compared to clinical assessments can be attributed to the measurement method, in which individual components of abduction/elevation are analyzed separately. As a result, total amplitudes appear lower, which is also reflected in normative reference values.

Flexion was only mildly reduced, with a mean of 135.1 ± 32.9 degrees on the affected side, compared to 145.3 ± 33.7 degrees on the unaffected side and 162.0 ± 11.8 degrees in the normative data.

**Figure 2:**
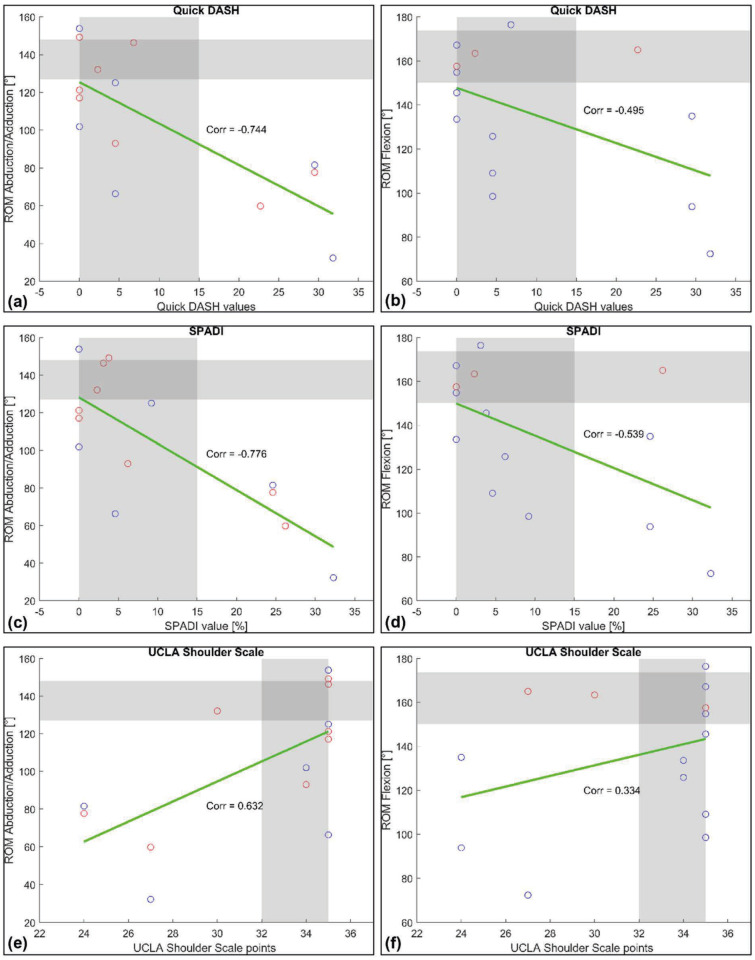
Correlation of postoperative active shoulder range of motion (ROM) with patient-reported outcome scores. Comparison of Quick-DASH scores to ROM in the frontal (**a**) and sagittal (**b**) plane. Comparison of SPADI scores to ROM in the frontal (**c**) and sagittal (**d**) plane. Comparison of UCLA Shoulder Scale scores to ROM in the frontal (**e**) and sagittal (**f**) plane. Blue circles indicate patients with trunk movement within normal limits (norm ± 2 SD): lateral trunk tilt in the frontal plane and back tilt in the sagittal plane. Red circles indicate increased tilt. Horizontal gray areas represent normative ROM ranges. Vertical gray areas indicate favorable score ranges. Green lines depict linear regression trends. Corr denotes Pearson’s product-moment correlation coefficient.

Pearson’s product–moment correlation coefficient was used to explore relationships between postoperative range of motion and functional scores (Quick-DASH, SPADI, UCLA Shoulder Scale), as illustrated in Figure 2.

Compensatory movements were predominantly observed in the frontal plane. Thoracic lateral tilting beyond normative values (mean ± 2 SD) was noted in 8 of 14 cases (57.1%). In the sagittal plane, compensatory movements were less frequent, occurring in only 3 of 14 patients (21.4%).

However, the presence of compensatory trunk movements did not affect subjective upper limb function or patient satisfaction.

## 4. Discussion

The present analysis of 15 cases treated with the modified Green procedure for Sprengel’s deformity demonstrated measurable improvements in both the clinical appearance and functional performance of the shoulder. Shoulder mobility in the frontal plane improved by an average of 55 degrees. The high functional capacity of the upper limb in daily activities was confirmed by favorable patient-reported outcome measures, with low Quick-DASH and SPADI scores and high UCLA Shoulder Scale values.

Three-dimensional motion analysis revealed compensatory movement patterns in more than half of the cases. These deviations did not negatively influence function or patient satisfaction, highlighting the clinical relevance and effectiveness of the procedure in improving quality of life in pediatric patients with high-grade deformity.

A scapulospinal connection was observed in 60% of cases, including a true osseous omovertebral connection in 40%, consistent with previous reports citing prevalence rates between 16% and 60% [3,5,6,7,26]. Sprengel’s deformity has been widely reported to be associated with a spectrum of congenital anomalies such as Klippel–Feil syndrome, spina bifida, scoliosis, rib anomalies, syringomyelia, and diastematomyelia [10,11]. Cardiovascular and urogenital anomalies may also be present, likely due to overlapping embryologic development phases [27]. In the present cohort, associated conditions were identified in 66.7% of cases, most frequently scoliosis (53.3%) and Klippel–Feil syndrome (26.7%). Öner et al. reported rib anomalies as the most frequent association (49%), followed by spina bifida (21%) and Klippel–Feil syndrome (21%) [27]. Di Gennaro et al. found scoliosis (48%) and Klippel–Feil syndrome (45%) as the most prevalent comorbidities, with some studies reporting Klippel–Feil syndrome in up to 82% of cases [28,29].

The Rigault and Cavendish classifications are well-established systems for assessing the severity of Sprengel’s deformity. All patients included in our cohort presented with high-grade deformities: Rigault grades 2 (60%) and 3 (40%), and Cavendish grades 3 (73.3%) and 4 (26.7%). According to a recent study by Antfang et al., surgical correction should be limited to Cavendish grades 3 and 4, as patients with milder forms often achieve satisfactory outcomes with conservative treatment [30]. Postoperative assessment showed significant aesthetic improvement, with 93.3% classified as Cavendish grade 1 and 6.7% as grade 2.

**Figure 3:**
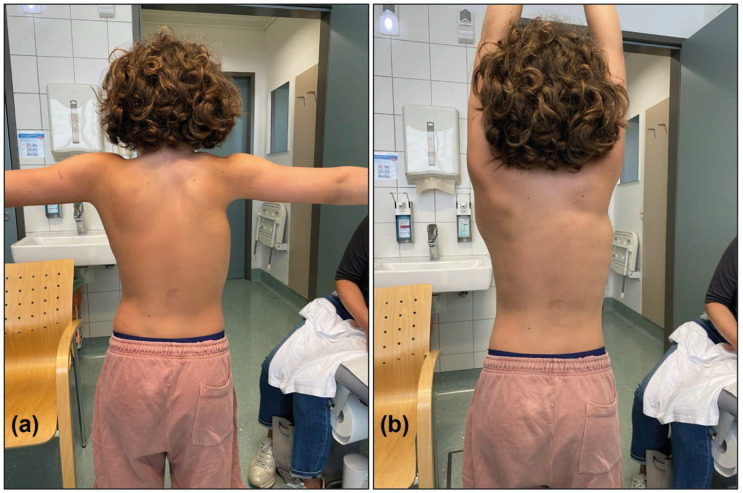
Postoperative shoulder function following the modified Green procedure. (**a**) Active abduction and (**b**) active elevation, illustrating excellent bilateral shoulder mobility following correction of the deformity on the right side.

In addition to improved appearance, significant functional gains were observed, with an average elevation of 163°, representing a 55° increase (Figure 3). Although the absolute postoperative increase in glenohumeral abduction appears limited, this finding must be interpreted in the context of the measurement definition used in this study. Glenohumeral abduction was assessed under manual scapular stabilization, enabling isolated evaluation of glenohumeral joint mobility, for which the maximal physiological range is 90°. Postoperative values of 90° therefore represent the maximal achievable range and can be considered an excellent outcome with respect to isolated glenohumeral mobility. When compared with previously published series, the magnitude of improvement in shoulder elevation observed in the present cohort is within the upper range of reported outcomes. Jiang et al. reported a mean elevation increase of 40°, with an average elevation of 142°, while Gonen et al. documented a gain of 44° to an average elevation of 145° [26,31]. Di Gennaro et al. observed only a 20° improvement to 112°, which may reflect the high rate of scoliosis and Klippel–Feil syndrome in their patient population [28].

One possible explanation for the comparatively larger functional improvement the patient group analyzed in this study is the routine use of prophylactic clavicular osteotomy in all cases. This may have allowed for greater intraoperative correction while minimizing neurovascular risks. Several authors have emphasized the benefits of clavicular osteotomy, especially in the absence of intraoperative neuromonitoring [4,6,16,32,33].

Notably, these results were achieved despite a relatively advanced mean age of 6.9 years at the time of surgery, compared to 4.5 to 4.8 years in other studies [26,31]. Although statistical significance was not reached, a trend toward improved outcomes in younger patients was observed, aligning with findings from Zarantonello et al. and Antfang et al. [17,30].

To date, this is the first report to utilize 3D motion analysis for evaluating postoperative upper limb movement patterns following surgical correction of Sprengel’s deformity. Deviations from normative patterns were observed in 57.1% of cases, particularly during abduction and elevation. These compensatory strategies appear to enhance functional performance and were not limited to patients with reduced mobility. The absence of correlation between altered movement patterns and shoulder range of motion suggests that these adaptations may be established preoperatively and persist after surgical correction.

Despite their presence, compensatory movements were not associated with limitations in daily function or decreased patient satisfaction, as confirmed by patient-reported outcome measures. All recorded scores indicated very good postoperative function and quality of life. The mean Quick-DASH score of 10.9 in this study is comparable to the value of 11.4 reported in a recent study by Antfang et al., which included six patients [30]. However, confirmation of these findings through larger cohorts is currently limited by the sparse data available in the literature and represents an important direction for future research.

Hypertrophic scarring remains a known consequence of the modified Green procedure due to extensive skin incisions, dissection, and scapular traction. In the present series, partial hypertrophic scar formation was observed in 40% of cases, consistent with previous reports citing rates from 22% to 64% [31,34,35,36]. Importantly, hypertrophic scarring was limited to scar widening and was not associated with pain, functional impairment, or keloid formation in any patient. Nonetheless, high satisfaction ratings for both function and cosmetic outcome (mean score: 8.9/10) underscore the clinical value of this surgical approach, even in light of its invasiveness.

Several limitations must be considered. The retrospective design and the relatively small sample size represent constraints. However, given the rarity of the condition and the complexity of its treatment, the sample size may still be considered adequate. The absence of preoperative patient-reported outcome measures limits interpretation to absolute postoperative scores, which, although reflecting favorable functional status and high patient satisfaction at final follow-up, do not allow quantification of subjective improvement.

Follow-up duration was heterogeneous as a consequence of the prolonged inclusion period required to assemble this cohort. Accordingly, outcomes should be interpreted as cross-sectional findings at the time of last follow-up rather than as longitudinal comparisons. It should be noted that postoperative physiotherapeutic treatment is primarily concentrated within the first six months after surgery, after which functional recovery largely stabilizes, allowing a meaningful assessment of postoperative function.

Finally, three-dimensional motion analysis relied on an adult reference dataset due to the lack of validated pediatric normative data. Although shoulder movement patterns and range of motion are largely comparable between children and adults, comparisons should be interpreted as exploratory and descriptive. Motion analysis data are also inherently dependent on patient compliance. This was addressed through a standardized protocol and averaging of five valid trials per assessment to reduce the risk of measurement bias.

## 5. Conclusions

The modified Green procedure appears to be a safe and effective surgical technique for the correction of Sprengel’s deformity in children, demonstrating favorable outcomes in terms of mobility, function, and quality of life in this cohort. A younger age at the time of surgery may serve as a positive predictor for improved postoperative outcomes. In the presence of preoperatively established compensatory movement patterns, postoperative persistence of these adaptations should be anticipated. However, their presence does not appear to compromise function or patient satisfaction. Given the retrospective design, small sample size, and lack of preoperative PROMs, these findings should be interpreted within the context of these methodological limitations.

## Figures and Tables

**Table 1:** Classification systems for grading the severity of Sprengel deformity.

Cavendish		Rigault	
Appearance of deformity with patient clothed		Superomedial angle of the scapula	
1	No visible deformity	1	Lower than T2 but above T4 transverse process
2	Prominence of superomedial angle	2	Between C5 and T2 transverse process
3	Shoulder asymmetry of 2–5 cm	3	Above C5 transverse process
4	Asymmetry > 5 cm with/without webbing		

**Table 2:** Frequencies and percentages of patient characteristics.

	Cases (*n* = 15), *n* (%)
Sex	
Male	10 (66.7%)
Female	5 (33.3%)
Scapula	
Hypoplastic	15 (100%)
Structural connection between scapula and spine	
Omovertebral bone	6 (40.0%)
Fibrous connection	3 (20.0%)
Associated congenital conditions	
Scoliosis	8 (53.3%)
Klippel–Feil syndrome	4 (26.7%
Other	6 (40.0%)
Rigault	
Grade 2	6 (40.0%)
Grade 3	9 (60.0%)

**Table 3:** Characteristics and frequencies (%) of Cavendish grades, shoulder abduction, and elevation pre- and postoperatively.

	Preoperative	Postoperative	*p*-Value
Cavendish			
Grade 1		14 (93.3%)	
Grade 2		1 (6.7%)	0.0001 ^1^
Grade 3	11 (73.3%)		
Grade 4	4 (26.7%)		
Abduction (°)			0.007 ^1^
*M* ± SD	82 ± 11	90 ± 0	
Range	60–90	90	
Elevation (°)			<0.0001 ^2^
*M* ± SD	108 ± 28	163 ± 25	
Range	85–170	90–180	

^1^ Wilcoxon signed-rank test, ^2^ Paired-samples *t*-test.

## Data Availability

The original contributions presented in the study are included in the article material, further inquiries can be directed to the corresponding author.

## Supplementary Materials

The following supporting information can be downloaded at https://www.mdpi.com/article/10.3390/jcm14248941/s1, Table S1: STROBE Checklist.
